# Investigations of the Tooth Pulp

**Published:** 1870-04

**Authors:** Franz Boll

**Affiliations:** Berlin Prussia


INVESTIGATIONS OF THE TOOTH PULP.—By Dr. Franz Boll, Berlin. Prussia.—(*Translated by Henriette Hirschfeld, D.D.S.*) (The April number of the *Dental Times*, of 1869, presented to its readers a short extract from an article published in the *Quarterly Journal of Microscopical Science*, entitled "Researches upon the Tooth Pulp," by M. Franz Boll, then medical student at Bonn. This extract contained the principal results of his investigations, and as these are of great importance to those interested or desirous of becoming conversant with dental histology, I feel justified in translating and communicating the whole article. At the same time I take great pleasure in introducing to my trans-atlantic professional brethren this young author. Since this was prepared he has graduated as a doctor of medicine, and has been made assistant to the celebrated Du Bois Raymond, Professor of Physiology at the University of Berlin. Professor Waldeyer, of the University of Breslau, eminent as a histologist and microscopist, says, in his splendid essay on the "Mouth and Development of Teeth," "we owe to Mr. Boll the first *definite knowledge of the condition* of the nerve fibres in the teeth." H. H.)

In the histology of tooth tissue I have directed my attention mainly to two points, the one being but slightly, and the other scarcely at all studied. I allude to the termination of the many nerve fibres entering into each tooth. The second—which has produced already a very extensive literature—treats of the proportions of the fundamental tooth substance to the tooth pulps, and the origin of the *first* out of the latter.

*The Termination of the Nerve Fibres.*—For the investigation of the nerve termination I can recommend as most suitable the long incisors of the rodents, guinea-pigs, rabbits, etc. The pulp cavity in animals not too old is quite large, and though the net-work of vessels and capillaries is also here extensively developed, it does not interfere as much as in the teeth of calves and sheep. The following results are obtained on the above mentioned objects:

If we take the incisors of an animal recently killed, crack it in a vice, extract the pulp, and apply a magnifying power of 200, it will not be difficult to distinguish the coarser anatomical features. The ramifications of a very rich net-work of vessels is to be seen, partly simple capillaries, partly more substantial arterial branches, with a layer of nonstriated muscular fibres, and a large number of thick, dark-bordered* nerve fibres, ascending parallel with the longitudinal axis of the pulp in fasciculi of six, eight, or more nerve fibrils. The larger vessels, as well as the nerve fibres, are enveloped in delicate fibrous connective tissue. The pulp does not contain other tissues beside these vessels and nerves; but in the interspaces left by them may be seen the cells of the embryonal pulp remaining, not used in this formation, though they have lost their spindle or spider-shaped form, so characteristic of the embryonical connective tissue.

The first reagent I used in the investigation of the nerves was hyperosmic acid. The weaker solutions of this are excellent in rendering visible the enormous quantity of dark-bordered nerve fibres, as well as the parallel arrangements of the thicker branches. The solutions are, however, of no use in examinations of the more minute ramifications of the dark-bordered nerve fibrils and their transition into the pale.

I expected much of that reagent by which Cohnheim made his important discovery of the free termination of the nerves of the cornea—the chloride of gold. This acts with wonderful effect on the cornea, as I can testify by my own experience, in making out the most minute nerve ramifications from the surrounding tissue. Hence I was first inclined to

---

* In the original the idea sought to be conveyed by "dark-bordered" and "pale" nerve fibres is represented by the words "markhaltig" and "marklos." It is impossible to translate these literally in this connection, but I think the original idea is fully carried out in the substitution.
J. T.

consider this reagent to be the true test for the nerve fibrils, but I am sorry to state that this is by no means the case. There is no coloring of the pulp even in the immersion of the pulps of small teeth. After several modified and unsuccessful experiments, I have abandoned this method and returned to the chromic acid, to which I still give the first rank for the investigations of the most delicate nerve ramifications. I take the pulp from an animal recently killed and place it in a small quantity of a weak solution (C 1-32 per ct.), and leave it there for nearly an hour, and investigate the specimen by pulling it apart in a drop of the same solution.

In examining a specimen obtained in this way, 500 times magnified, we perceive an enormous quantity of most minute fibrils of a peculiar silky appearance, besides very many dark-bordered ones splendidly marked by this method. These fibrils might, at the first glance, be taken for elastic fibres of the most delicate kind, if they did not, with great probability, present themselves as fine, pale, nerve fibrils. The transition of the dark bordered into the pale fibrils is very gradual. First the axis cylinder is still enveloped in a comparatively thick sheath, which exhibits distinctly double contours. The medullary part is diminished, the granulations of the myelin are concentrated only at certain parts, where they form the characteristic varicoses. These, also, have double contours, while the extremely thin layer of nervous matter which surrounds the axis cylinder between each two varicoses exhibits only simple contours. At first the varicoses are very close together, and the spaces between them very small, but these latter soon become larger, while the former are less frequent, and soon entirely disappear. Although the fibrils then become very delicate, their varying diameter can be still distinguished. These markings are also soon lost, and they appear as bare, simple, homogeneous axis cylinders. These finest nerve fibrils show, as above mentioned, a certain similarity to elastic fibres, but their very great delicacy, and the little resistance they show to the reagents, prevent their being confounded with the former. By adding water or acetic acid they enlarge directly and disappear. Treated with a cold concentrated solution of oxalic acid they become beautifully distinct for a time. Shortly these bare axis cylinders commence to exhibit faint but distinct varicoses, and after one or two days they likewise have become enlarged and have disappeared. Another convincing proof of their nervous character is, to my mind, their very frequent but always dichotomous division, while an anastomotic connection of two branches, as is so frequently found with elastic fibres, has never been observed. Moreover, the course is generally straight, very seldom undulating like the elastic fibres.

In the investigation of the termination of these delicate, pale nerve fibrils, we are met by quite a peculiar difficulty. It will be found that in applying the usual method, *i. e.* cracking the fresh tooth in a vice and removing the pulp with a fine pincer, we never secure the *whole* pulp, or *all* the soft parts of it. The superficial layer of the pulp, constituting the boundary toward the walls of the cavity formed by the substantia eburnia (dentine), consists of a simple continuous layer of longitudinal cells, which, by their long processes projecting into the tubes of the substantia eburnia, are fastened, as it were, to it. Let one be ever so careful in extracting the pulp from the freshly cracked tooth, a trace of this peculiar layer will rarely be found on the surface of the pulp. Kept in close connection to the walls of the dentine, by penetrating into its tubes with their projections, they will appear to the naked eye as a thin slimy covering. By scraping it carefully with a fine knife and placing it under the microscope, it will be observed to consist also, beside this most superficial layer, of another layer of cells, forming the connection with the pulp tissue proper. Now, to obtain the whole pulp perfect, I proceed in the following manner: I crack the fresh tooth *once* in the vice, and place it immediately—pulp and tooth substance still in connection—in the chromic acid solution. After the lapse of an hour I carefully remove a portion of the loose pieces of the tooth, and then try to insert a very sharp knife between the pulp and the dentine. With some good luck and experience one may succeed in this way in obtaining the superficial layers of the pulp *in continuo*. The peripheral processes which are inclosed in the dentinal tubes generally break close to the body of the cell, but by a skillful movement of the knife they may sometimes be successfully extracted of considerable length from the dental tubes. The more striking feature of the specimens thus obtained is the dichotomous division of enormously increased quantity of pale fibrils. In pulling it apart with the most delicate needles hardly a piece will be seen without some of these minute fibrils. At the boundary of the vesicular pulp tissue proper and the superficial cell-layers is an especial dense net work to be seen, usually remaining fastened to the wall of the pulp cavity. By perseverance some may be procured from among those pulled pieces on which the superficial layers are preserved *in situ*. The proportions of the superficial layers of the pulp, as well as the terminations of the nerve fibrils, present themselves in perfect profile. From the net-work beneath the superficial cell-layer may be observed some delicate nerve fibrils ascending vertically, and finding their way to the free surface of the pulp, between the cells, which are finally nearly in close contact with each other. On the surface they project considerably above the cells. I have drawn two such specimens. The first one is especially instructive, though the projections of the superficial cells, inserted in the dentinal tubes, are torn near the body of the cells, but above the surface the projecting nerve terminations are preserved and the better to be seen. In the second specimen though not as perfect as the first case, the peripheral processes are all preserved, but it is there possible to exhibit the nerve fibrils projecting between them.

It is now left to elucidate the question: Do these free, projecting ends of the nerve fibrils penetrate into the dentinal tubes or not? I have taken great pains to give undoubted evidence thereof, but have not succeeded satisfactorily. I placed the fresh teeth in chromic acid of about 1-32 per ct., and concentrated this solution gradually, until, at the expiration of some weeks, it contained 2 per cent. and over. This strong solution had so decalcified them in from five to six weeks, that I was enabled to make thin microscopic sections, without, however, obtaining specimens to give indisputable proofs. Just at the boundary of the substantia eburnia and the pulp was always a dim, granulated layer rendering a definite decision impossible. Decalcification by weak solutions of nitric or muriatic acid, after a previous hardening in bichromate of potassa, proved still worse. The effect of this was to destroy the nerve fibrils. In spite of the lack of decided proof, I am, nevertheless, perfectly satisfied that the nerve fibrils penetrate into the dentinal tubes.

Beside the merely physiological reason—that of the well known sensitiveness of dentine—I am also enabled to give anatomical reasons for this assertion. The surface of the superficial cell-layer of the pulp and the inner wall of the pulp cavity are in such close contact, that there would be no room for the free rising nerve terminations. Moreover, the direction of the fibrils is so exactly parallel with the dentinal tubes that we can hardly suppose anything else but of their being extracted from them, like the processes of the pulp cells. We will, therefore, be justified in the conclusion, that there are two kinds of tubes in that part of the dentine nearest to the pulp, one part which receives the processes of the superficial cells, and another to receive the minute nerve fibrils.

[To be continued.]

